# Generation of an iPSC line from a Pontocerebellar Hypoplasia 1B patient harboring a homozygous c.395 A > C mutation in *EXOSC3* along with a family matched control

**DOI:** 10.1016/j.scr.2022.102944

**Published:** 2022-10-13

**Authors:** Ben N. Stansfield, Sampath Rangasamy, Keri Ramsey, May Khanna, Jared M. Churko

**Affiliations:** a Department of Cellular and Molecular Medicine, University of Arizona, Tucson, AZ, USA; b Sarver Heart Center, University of Arizona, Tucson, AZ, USA; c Department of Physiology, University of Arizona Health Sciences, Tucson, AZ 85724, USA; d TGen, Phoenix, AZ 85004, USA; e Department of Pharmacology, College of Medicine, University of Arizona, Tucson, AZ 85724, USA; f Center for Innovation in Brain Science, Tucson, AZ 85721, USA

## Abstract

Pontocerebellar Hypoplasia 1B (PCH1B) is a severe autosomal recessive neurological disorder that is associated with mutations in the exosome complex component RRP40 (*EXOSC3*) gene. We generated and characterized an iPSC line from an individual with PCH1B that harbors a recessive homozygous c.395 A > C mutation in *EXOSC3* and a family matched control from the probands unaffected mother. Each iPSC line presents with normal morphology and karyotype and express high levels of pluripotent markers. UAZTi009-A and UAZTi011-A are capable of directed differentiation and can be used as a vital experimental tool to study the development of PCH1B.

## 1. Resource utility

Two iPSC lines generated allow for the generation of various cell types implemented in Pontocerebellar Hypoplasia 1B which will enable researchers to study the c.395 A > C mutation in *EXOSC3* and the mechanisms by which it contributes to the disease phenotype (Table 1).

## 2. Resource details

**Fig. 1.**
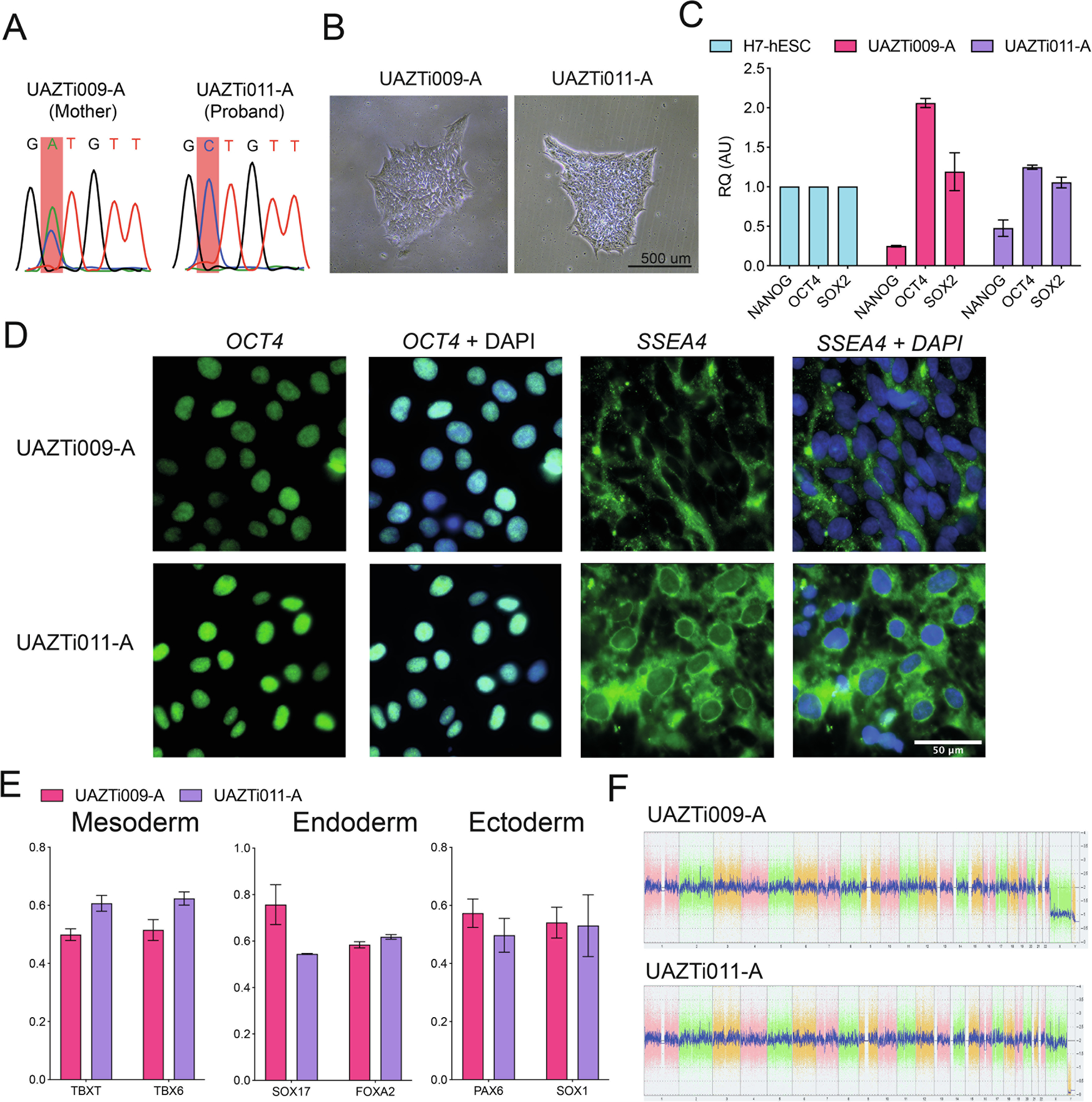
Characterization of UAZTi009-A and UAZTi011-A iPSC lines.

Pontocerebellar Hypoplasia 1B is a severe autosomal recessive neurological disorder and is clinically characterized by large neuronal loss in the ventral pons and inferior olive, motor neuron degeneration in the spinal cord, and underdevelopment of the neocerebellum (Rudnik-Schoneborn et al., 2013). Exosome complex component RRP40 (*EXOSC3*) is a subunit of the RNA exosome complex and mutations in *EXOSC3* are associated with Pontocerebellar Hypoplasia 1B and neuro degeneration (François-Moutal et al., 2018). Using Sendai virus reprogramming (Churko et al., 2013) we have generated one iPSC line from fibroblasts of a Pontocerebellar Hypoplasia 1B patient harboring the homozygous c.395 A *>* C, mutation in *EXOSC3* (Fig. 1A), and an iPSC line from their unaffected mother. All cell lines possess normal morphology (Fig. 1B) and were negative for mycoplasma (Supplementary Fig. 1A) and Sendai virus (Supplementary Fig. 1B). All iPSC lines expressed the iPSC markers *SOX2*, *NANOG* and *OCT4* at the mRNA level and *OCT*4, and *SSEA4* at the protein level assessed by reverse transcription quantitative polymerase chain reaction (RT-qPCR) (Fig. 1C) and immunocytochemistry (Fig. 1D) respectively. iPSC lines were able to undergo directed differentiation into the three germ layers and were positive for mRNA markers of Endoderm, Ectoderm and Mesoderm when differentiated (Fig. 1E). Furthermore, no karyotypic abnormalities were observed (Fig. 1F).

## 3. Materials and methods

### 3.1. Reprogramming

Reprogramming of patient fibroblasts was performed at the University of Arizona iPSC core under IRB approval. Fibroblasts were isolated from skin puncture biopsies and 250,000 cells were incubated with Sendai virus to express Sox2, Oct3/4, klf4 and cMyc using the Cyto-Tune®-iPS v2.0 Reprogramming Kit (Thermo Fisher Scientific; A16517). After ∼ 20 days, single iPSC colonies were picked and expanded in Essential 8 (E8) medium (Thermo Fisher Scientific).

### 3.2. Cell Culture

iPSC lines were cultured in E8 media on 6 well plates coated with growth factor reduced Matrigel (Corning™; Cat #CB356238) until 70 % confluency under normoxic conditions (37°C, 5 % CO_2_, 20 % O_2_). iPSC lines were passaged in E8 and 10 μM ROCK inhibitor (Y27632, Tocris; #Cat1254) at a 1:6 ratio every 4–5 days.

### 3.3. Mutation analysis

DNA was extracted and purified from each iPSC line before PCR amplification using PrimeStar GXL polymerase (Takara; R050B) according to manufacturer’s instructions. Amplicons were sequenced for the c.395 A > C mutation in *EXOSC3* (Eton Biosciences) to confirm the genotype of each individual cell line by sanger sequencing.

### 3.4. Immunocytochemistry

iPSC lines were seeded onto Matrigel coated coverslips and cultured in E8 medium until fixation. Cells were fixed in 3.7 % formalin for 60 min at room temperature. Fixed cells were permeabilized with 0.15 % Triton X-100 in PBS prior to blocking in 1 % BSA, 22.52 mg/ml glycine and 0.1 % Tween 20 in PBS (PBST). Cells were incubated with primary antibodies (Table 2) in blocking solution (minus glycine) overnight in a humidified chamber at 4 °C. Following the primary antibody incubation, secondary antibodies (Table 2) were incubated at room temperature in the dark for 60 min. Following secondary antibody incubations, cells were washed 3 × 5 min in PBST prior to nuclear counter staining using DAPI (ThermoFisher; D1306).

### 3.5. Trilineage Differentiation

All iPSC lines were differentiated into Endoderm, Ectoderm and Mesoderm using the StemMACS™ Trilineage Differentiation Kit (Miltenyi Biotech; 130-115-660) according to manufacturer instructions.

### 3.6. RT-qPCR

RNA was extracted and isolated from iPSCs at passage 15–18 using the Direct-zol RNA Miniprep kit (Zymo Research; R2052). Isolated RNA was reverse transcribed into cDNA using the SuperScript VILO master mix (ThermoFisher; 11755050). qPCR was performed using the power track SYBR Green Master Mix (ThermoFisher; A46012). Samples were normalized to the H7 hESC line (WiCell).

### 3.7. Mycoplasma testing

Mycoplasma analysis was conducted using the PCR Mycoplasma Test Kit I/C (PromoKine, PK-CA91-1024) using the high sensitivity method, according to manufacturer instructions.

### 3.8. Karyotyping and STR analysis

2 × 10^6^ cells were collected from each line at passage 15–17 and karyotyping analysis was conducted by Life Technologies using the KaryoStat^+^™ assay (ThermoFisher). Primary human fibroblast samples were used for STR analysis to confirm genetic similarity between patient fibroblasts and iPSC lines.

### 3.9. Sendai virus Clearance

RNA was collected from cells at passage 15–16 using the the Directzol RNA Miniprep kit (Zymo Research; R2052). RNA was amplified by PCR using Taq DNA polymerase (GoldBio, T-514). Cells at passage 1 were used as a positive control.

## Supplementary Material

Supplementary Figure

## Figures and Tables

**Table 1:** Characterization and validation.

Classification	Test	Result	Data

Morphology	Bright field	Normal	Fig. 1Panel B
Phenotype	Qualitative analysis. Immunocytochemistry	Assess staining of pluripotency markers; *OCT4*, *SSEA*-4	Fig. 1Panel D
	Quantitative analysis; RT-qPCR	Assessed the Pluripotency markers *OCT4*, *NANOG*, *SOX2*	Fig. 1Panel C
Genotype	Karyotype (KaryoStat) *150 k SNPs analysed*>2 Mb (Chromosomal gains) >1 Mb (Chromosomal losses)	Normal	Fig. 1 Panel F
Identity	Microsatellite PCR (mPCR) OR STR analysis	Not performed Normal	NA
Mutation analysis (IF APPLICABLE)	Sanger Sequencing	UAZTi009-A; Heterozygous Mutation UAZTi011-A; Homozygous Mutation	Fig. 1 Panel A
	Southern Blot OR WGS	NA	NA
Microbiology and virology	Mycoplasma	Mycoplasma Negative; Tested by PCR	Supplementary Fig. 1A
Differentiation potential	Directed differentiation	Directed Differentiation into Ectoderm, Endoderm and Mesoderm germ layers	Fig. 1 Panel E
List of recommended germ layer markers	RT-qPCR	Markers Assessed via RT-qPCR Ectoderm: *PAX6*, *SOX1* Endoderm: *SOX17*, *FOXA2* Mesoderm: *TBXT*, *TBX6*	Fig. 1 Panel E
Donor screening (OPTIONAL)	HIV 1 + 2 Hepatitis B, Hepatitis C	N/A	N/A
Genotype additional info (OPTIONAL)	Blood group genotyping	N/A	N/A
	HLA tissue typing	N/A	N/A

**Table 2:** Reagents details.

	Antibodies used for immunocytochemistry/flow-cytometry			
	Antibody	Dilution	Company Cat #	RRID

*Pluripotency Markers (ICC)*	*Mouse anti-OCT4*	1:100	#60093	AB_2801346
	*Mouse Anti SSEA4*	1:100	#560308	AB_1645371
*Secondary antibodies*	F(ab’)2-Goat anti-Mouse IgG (H + L) Cross-Adsorbed Secondary Antibody, Alexa Fluor 488	1:1000	#A11017	AB_2534084
	Primers Target	Size of Band	Forward/Reverse primer (5′–3′)	
Pluripotency Markers (qPCR)	*NANOG*	116	TTTGTGGGCCTGAAGAAAACT/AGGGCTGTCCTGAATAAGCAG	
	*OCT4*	106	CCTGAAGCAGAAGAGGATCACC/AAAGCGGCAGATGGTCGTTTGG	
	*SOX2*	150	AGAAGAGGAGAGAGAAAGAAAGGGAGAGA/GAGAGAGGCAAACTGGAATCAGGATCAAA	
Differentiation Markers; Ectoderm (qPCR)	*PAX6*	131	CTGAGGAATCAGAGAAGACAGGC/ATGGAGCCAGATGTGAAGGAGG	
	*SOX1*	136	GAGTGGAAGGTCATGTCCGAGG/CCTTCTTGAGCAGCGTCTTGGT	
Differentiation Markers; Endoderm (qPCR)	*SOX17*	112	ACGCTTTCATGGTGTGGGCTAAG/GTCAGCGCCTTCCACGACTTG	
	*FOXA2*	134	GGAACACCACTACGCCTTCAAC/AGTGCATCACCTGTTCGTAGGC	
Differentiation Markers; Mesoderm (qPCR)	*TBXT*	153	CCTTCAGCAAAGTCAAGCTCACC/TGAACTGGGTCTCAGGGAAGCA	
	*TBX6*	136	TCATCTCCGTGACAGCCTACCA/CCGCAGTTTCCTCTTCACACGG	
House Keeping Gene(s)	*18 s rRNA*	159	ACCCGTTGAACCCCATTCGTGA/GCCTCACTAAACCATCCAATCGG	
Targeted mutation	*EXOSC3*	941	CCTGTCCCTCCTCTAAGCCT/ACGTTTGGAGACCGTGAGTC	
Sendai Virus Clearance	*SeV*		CAGAGGAGCACAGTCTCAGTGTTC/TCTCTGAGAGTGGTGCTTATCTCTGT	

RRID Requirement for antibodies: use https://antibodyregistry.org/ to retrieve RRID for antibodies and include ID in table as shown in examples.

**Resource Table:** 

Unique stem cell lines identifier	1) UAZTi009-A 2) UAZTi011-A
Alternative name(s) of stem cell lines	UAZTi009-A; MKAZ1 UAZTi011-A; MKAZ3
Institution	University of Arizona
Contact information of distributor	Dr Jared Churko PhD jchurko@arizona.edu
Type of cell lines	iPSC
Origin	Human
Additional origin info required for human ESC or iPSC	UAZTi009-A; Female UAZTi011-A; Male
Cell Source	Fibroblast
Clonality	Clonal
Method of Reprogramming	Sendai Virus
Sendai Virus Clearance	PCR
Type of Genetic Modification	NA
Cell Culture System	Matrigel
Associated disease	Pontocerebellar Hypoplasia 1B
Gene/locus	*EXOSC3* c.395 A > C
Date archived/stock date	May 31, 2022
Cell line repository/bank	https://hpscreg.eu/cell-line/UAZTi009-A https://hpscreg.eu/cell-line/UAZTi011-A
Ethical approval	WIRB® Protocol #20120789, and University of Arizona #1808846797

## Abbreviations:

EXOSC3: Exosome Component 3
