# Supplementary figures and images for: Generation of an iPSC line from a Pontocerebellar Hypoplasia 1B patient harboring a homozygous c.395 A > C mutation in EXOSC3 along with a family matched control
Source: Stem Cell Res. Author manuscript; Available in PMC 2022 Dec 8. (PMC9729447; doi:10.1016/j.scr.2022.102944)

# Supplementary Figure 1

A

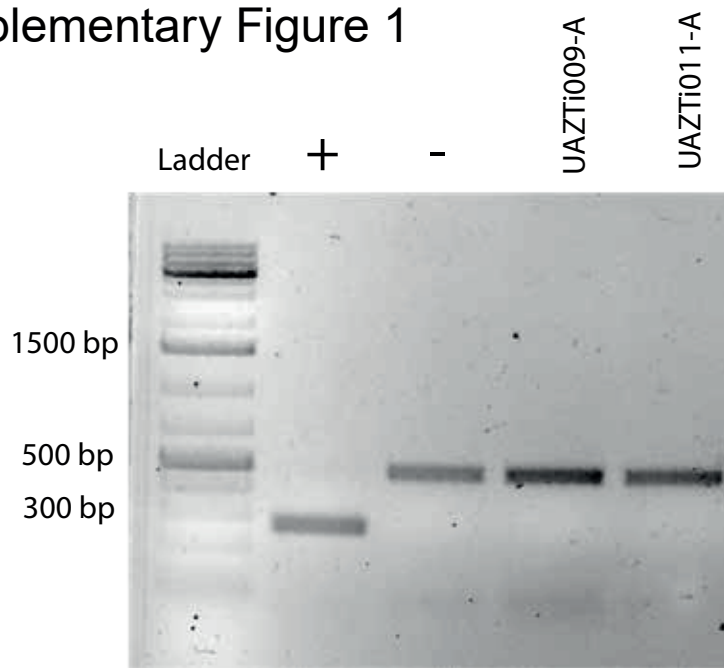

B

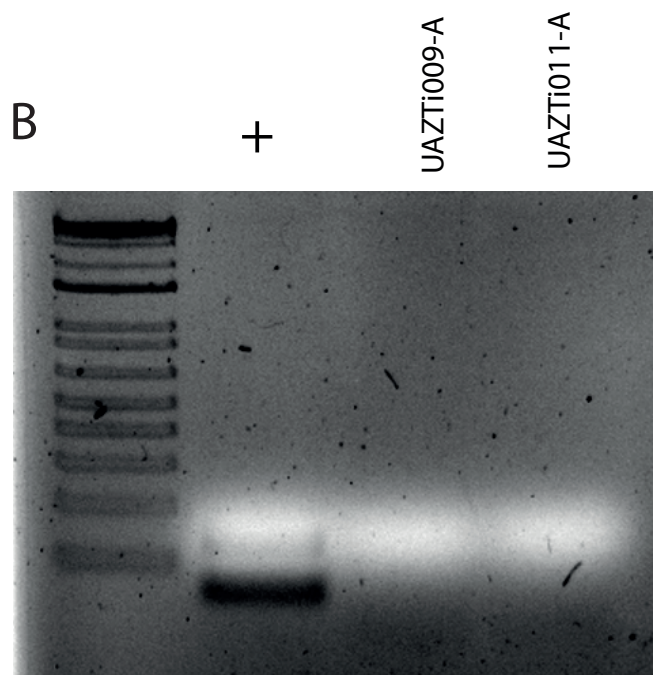

Supplement: Supplementary Figure [file NIHMS1846233-supplement-Supplementary_Figure.pdf]
